# Prevalence, perception and factors associated with diabetes mellitus among the adult population in central Vietnam: a population-based, cross-sectional seroepidemiological survey

**DOI:** 10.1186/s12889-017-4208-9

**Published:** 2017-04-05

**Authors:** Masami Miyakawa, Takayuki Shimizu, Nguyen Van Dat, Phung Thanh, Pham Thi Phuong Thuy, Nguyen Thi Hoang Anh, Nguyen Huu Chau, Yumi Matsushita, Hiroshi Kajio, Vien Quang Mai, Masahiko Hachiya

**Affiliations:** 1 Bureau of International Health Cooperation, National Center for Global Health and Medicine, 1-21-1, Toyama Shinjuku-ku, Tokyo, Japan; 2 Pasteur Institute Nha Trang, 8-10 Pasteur, Xuong Huan, tp, Nha Trang, Khánh Hòam Vietnam; 3 Endocrinology Center, Khánh Hòa Provincial Public Health Service, 3A Han Thuyen Street, Nha Trang City, Khánh Hòa Province Vietnam; 4 Center for Clinical Sciences, National Center for Global Health and Medicine, 1-21-1, Toyama Shinjuku-ku, Tokyo, Japan; 5 Department of Diabetes, Endocrinology and Metabolism, Center Hospital, National Center for Global Health and Medicine, 1-21-1, Toyama Shinjuku-ku, Tokyo, Japan

**Keywords:** Diabetes mellitus, Central Vietnam, Prevalence, Perception

## Abstract

**Background:**

Diabetes Mellitus (DM) has rapidly become a major public health concern in Vietnam. Although the prevalence of DM has been studied in northern and southern Vietnam, little data are available for the central region. Therefore, the aims of this survey were to estimate the prevalence of DM and to identify the perception of and factors associated with DM among the adult population in central Vietnam.

**Methods:**

We conducted a cross-sectional, population-based survey in Khánh Hòa Province, Vietnam in December 2014 using three-stage cluster sampling and probability proportional to size sampling in line with the World Health Organization STEPwise approach. Four hundred and eighty residents aged 20–70 years were selected from 30 villages in 10 wards/communes. After obtaining informed consent, all residents participated in interviews regarding lifestyle, medical history, and perception of DM and underwent physical measurements and blood examination for fasting blood glucose and glycated hemoglobin. Factors associated with DM were analyzed using a logistic regression model.

**Results:**

A total of 376 residents were enrolled (response rate: 78.3%; females: 59%; rural residents: 61%). Among the participants, 14.3% and 18.9% of males and females, respectively, were classified as overweight/obese according to body mass index (BMI), 37.7% and 22.1%, respectively, had hypertension, and 36.4% and 11.7% had metabolic syndrome. The prevalence of DM in the entire population was 7.2% (27/376; 95% confidence interval [CI]: 4.6–9.8). Participants aged 60–70 years were more likely to have DM than those aged 30–39 years (adjusted odds ratio [aOR]: 8.7; 95%CI: 1.4–56.0), and participants classified as obese were more likely to have DM than those with normal or low BMI (aOR: 10.2; 95%CI: 2.2–50.2). Furthermore, more than two-thirds (254/376, 67.6%) of the participants either did not understand or had never heard of DM, and less than half of the DM cases (12/27, 44%) were aware of their history of DM.

**Conclusions:**

The results of this study suggested that the prevalence of DM among the adult population in central Vietnam was slightly higher than that in other areas. Additional research is needed to further explore perceptions of and practices regarding DM.

## Background

Diabetes mellitus (DM) is a major noncommunicable disease that has rapidly become a major public health concern around the world. In 2014, the number of the adult population with DM had increased to 422 million (prevalence: 8.5%), with a particularly sharp rise in middle- and low-income countries. DM was the direct cause of 1.5 million deaths in 2012 [1, 2] and is projected to be the seventh leading cause of death by 2030 [3].

In Vietnam in 2015, 3.5 million adults aged 20–79 years were estimated to have had DM, 1.8 million of whom remaining undiagnosed; in addition, DM was responsible for 53,400 deaths [4]. Recent studies have reported that the prevalence of DM in the adult Vietnamese population from 2004 to 2016 was 3.7–6.0% (95% confidence interval [CI]: 2.7–6.7%), with 4.7–10.8% for males and 5.0–11.7% for females [4–9].

The following factors have also been reported as being associated with DM in Vietnam: classification as being overweight/obese according to body mass index (BMI) in 11.1–19.5% of males and 14.1–28.3% of females [5, 10], hypertension in 27.3% of males and 16.2% of females [10], and metabolic syndrome in 13.9% of males and 18.5% of females [11].

In addition, the following have been identified as risk factors for DM in other studies in Vietnam: being female, older, or overweight/obese, having hypertension or a familial history of DM, engaging in light physical activity, or living in an urban area [6–9].

Other studies reported that 40–73% of the cases had been unaware that they had DM [7, 9].

Although the prevalence of DM has been studied in northern and southern Vietnam, as described above, little data are available for the central region. Therefore, we conducted a survey in this region to estimate the prevalence of DM and to identify the perception of and factors associated with DM in central Vietnam.

## Methods

### Setting

We conducted a cross-sectional, population-based seroprevalence survey in Khánh Hòa Province, which is in the south-central coastal region of Vietnam, in December 2014 using three-stage cluster sampling. This province covers an area of 5217.7 km^2^ and had a population of nearly 1.2 million as of 2014 [12]. The primary economic sectors of this province are industry, construction, and services-tourism [13], and according to official provincial documents 56% of the communities are rural.

### Subjects and sampling methods

We targeted adult residents of Khánh Hòa Province aged 20–70 years; pregnant women or those who had given birth within the previous 3 months were excluded in order to avoid misclassification due to the likelihood of unstable glycosylated hemoglobin (HbA1c) levels caused by gestational DM.

The required sample size was 426.8 as determined using the equation n = *Z* ^2^ × *p*(1-*p*)*Deff*/(*e* ^2^ × *RR*) from the sample size calculator [14] provided by the World Health Organization (WHO) STEPwise approach to noncommunicable disease risk factor surveillance (STEPS) [15] with a level of confidence (*Z*) of 1.96 for a 95% CI, an estimated prevalence of DM (*p*) of 0.05, a design effect (*Deff*) of 2.0, a margin of error (*e*) of 0.03, and an expected response rate (*RR*) of 0.95. A total of 480 subjects were randomly selected from the population and residents lists from the province using probability proportional to size (PPS) sampling as follows: 10 out of 137 wards/communes, three villages from each ward/commune, and 16 adult inhabitants from each village.

### Case definitions

Blood was collected after at least 8 h of fasting, as instructed in invitation letters to the participants. Those who met any of the following criteria were consider to have DM [16]: 1) elevated HbA1c level ≥ 6.5%; 2) elevated fasting plasma glucose (FPG) level ≥ 7.0 mmol/L (126 mg/dL) or random elevated plasma glucose level ≥ 11.1 mmol/L (200 mg/dL); or 3) history of treatment for DM (lifestyle guidance including diet or exercise advice, oral medication, or insulin). Those with a marked discrepancy between HbA1c and FPG levels without factors related with DM who were strongly suspected as having hemoglobinopathy were excluded based on clinical grounds.

Residential areas were categorized as either rural or urban based on government classifications. Monthly household income was converted according to the rates in December 2014 (1,000,000 VND = 47.3 USD) [17]. BMI was calculated as body weight (kg) divided by the square of height (m^2^), and then classified as follows: overweight as ≥25 kg/m^2^ and <30 kg/m^2^, and obese as ≥30 kg/m^2^ [18]. Hypertension was defined as elevated blood pressure (BP), with systolic BP ≥ 140 mmHg and/or diastolic BP ≥ 90 mmHg [19]. Hyperlipidemia was determined as the existence of any of the following factors: 1) high total cholesterol (TC) level as ≥6.2 mmol/L (240 mg/dL) from a multi-country study [20]; 2) low high-density lipoprotein cholesterol (HDL-C) level as <1.0 mmol/L (40 mg/dL); and 3) high triglyceride level as ≥2.3 mmol/L (200 mg/dL) based on the definition of the laboratory institute. Metabolic syndrome was defined as the existence of any three of the following five factors: 1) waist circumference ≥ 90 cm for males or ≥80 cm for females in the Asian population; 2) triglyceride level ≥ 1.7 mmol/L (150 mg/dL); 3) HDL-C level < 1.0 mmol/L (40 mg/dL) for males and <1.3 mmol/L (50 mg/dL) for females; 4) systolic BP ≥ 130 mmHg and/or diastolic BP ≥ 85 mmHg; and 5) FPG level ≥ 5.6 mmol/L (100 mg/dL) or drug treatment for elevated glucose level [21].

### Perception and self-reported history of DM in Khanh Hoa, Vietnam

We also explored the perception of DM using structured questionnaires by asking whether they had heard of it or not, moreover whether they understood what DM was if they had heard of it before. Likewise we asked their recognition of current and/or past history of high blood sugar values or DM to let them choose one among yes, no, or unknown. These answers were self-reported and their actual knowledge regarding DM were not verified, while the status of DM in each subject were determined in this study the results of which were tabulated.

### Survey process

The survey process and procedures were carried out in reference to STEPS [15].

Two survey teams composed of researchers or medical and paramedical officers from the Nha Trang Pasteur Institute (IPN) and the Endocrinology Center of Khánh Hòa Provincial Health Service were employed, with three supervisors and nine surveyors deployed to each team. A 2-day training session on the survey process, including questionnaire pretesting, was conducted prior to the survey. General information regarding the survey was sent in an announcement letter to the participants, accompanied by instructions to fast for at least 8 h before blood tests.

The survey was conducted at 10 commune health centers (CHCs) over a 6-day period. Participants living in the three villages in each catchment area were invited to their respective CHCs at dawn, and the survey process was conducted throughout the early morning. Laboratory tests and data entry were then carried out in the afternoon every day at the IPN.

After obtaining written informed consent, interviews were conducted using a structured questionnaire, BP and anthropometric measurements were taken, and blood sampling was performed. The structured questionnaire was composed of closed questions with regard to sociodemographic profiles, lifestyle activities, history and perception of DM, and time of last food intake to assure proper fasting prior to testing for FPG. Anthropometric measurements included height, weight, and waist and hip circumference. Venous blood samples (5 mL) were drawn from each participant by experienced nurses or lab technicians and collected into ethylenediaminetetraacetic acid (EDTA) or plain blood tubes. The EDTA tubes were stored in special blood containers (CubeCooler, Forte Grow Medical Co., Ltd., Tochigi, Japan) at 0–5 °C. Blood in plain tubes was centrifuged at 3000 rpm for 15 min within 30 min after sampling, and supernatant serum was then transferred into microtubes stored on ice to prevent degradation of glucose levels. Finally, all questionnaires were checked by supervisors to ensure that all required answers were appropriately marked.

### Blood tests

Blood samples processed in each CHC were transported to the IPN within about 6 h after sampling and tested for HbA1c, glucose, TC, HDL-C and triglycerides. HbA1c was quantified using high-performance liquid chromatography (HPLC; HLC-723 G8; TOSOH, Tokyo, Japan). Glucose, TC, HDL-C and triglycerides were measured in serum. The type of DM among those determined to have DM in this study were not differentiated by further detailed investigation.

### Statistical analysis

All data were validated using double-entry methods and data cleaning, and then analyzed using STATA IC 11 (Stata Corp., College Station, TX, USA) in accordance with the case definitions as indicated above. The characteristics of the enrolled participants were tabulated, and the proportions of those who had DM were calculated using point estimation with 95%CIs. Odds ratios (ORs) and adjusted ORs (aORs) of having DM (95%CI) were estimated in univariate and multivariate analysis using a logistic regression model. The association was regarded as being statistically significant when the 95%CI did not contain the value 1.0.

### Ethical considerations

This study was approved by the Institutional Review Boards of the National Center for Global Health and Medicine (NCGM-G-001644-00) in Japan and the IPN. In addition, the protocol and operation of this survey were authorized by the Khánh Hòa Province People’s Committee and the Provincial Health Department. Informed consent was obtained in writing or by thumbprint from all participants. All personal information was kept confidential. The notice of results included advice for medical consultations at CHCs or other health facilities if any abnormal findings were found, and the CHCs involved in this study were expected to follow-up the participants accordingly.

## Results

### Characteristics of the participants

As shown in Table 1, 376 of the 480 participants were finally enrolled (response rate: 78.3%). The reasons for failed enrollment included moving out of the study area or refusing to participate. Females accounted for 59.0% of the participants, and the mean age ± standard deviation was 42.9 ± 12.7 years. Rural residents accounted for 61.4% of the participants and tended to be younger than urban residents. More than half of the males were manual workers, with more than 60% engaging in heavy physical activity. Regarding females, three in 10 were homemakers or unemployed, with more than 60% engaging in light activity. Nearly 80% of males were current or past smokers and 13% drank alcohol more than 4 days per week. By contrast, only a small proportion of females engaged in such behaviors. Among the participants, 14.3% and 18.9% of males and females, respectively, were classified as overweight/obese according to BMI, 37.7% and 22.1% as having hypertension, and 36.4% and 11.7% as having metabolic syndrome.

**Table 1:** Characteristics of and prevalence of diabetes mellitus among the enrolled participants in Khanh Hoa, Vietnam

Factor	Category	Males		Females	
		(*n* = 154)		(*n* = 222)	
		Total n (%)	DM^a^ n (%)	Total n (%)	DM^a^ n (%)
Total		154 (41.0)	11 (7.1)	222 (59.0)	16 (7.2)
Age (years old)	20–29	36 (23.4)	0 (0.0)	36 (16.4)	0 (0.0)
	30–39	32 (20.8)	1 (3.1)	52 (23.6)	1 (1.9)
	40–49	33 (21.4)	3 (9.1)	57 (25.9)	4 (7.0)
	50–59	39 (25.3)	5 (12.8)	51 (23.2)	4 (7.8)
	60–70	14 (9.1)	2 (14.3)	24 (10.9)	6 (25)
Residential area	Rural	103 (66.9)	3 (2.9)	128 (57.7)	6 (4.7)
	Urban	51 (33.1)	8 (15.7)	94 (42.3)	10 (10.6)
Occupation	Manual worker	86 (55.8)	2 (2.3)	67 (30.2)	4 (6)
	Office worker	16 (10.4)	2 (12.5)	23 (10.4)	1 (4.4)
	Service worker	20 (13)	5 (25)	41 (18.5)	1 (2.4)
	Homemaker, Student, No job	9 (5.8)	0 (0)	69 (31.1)	6 (8.7)
	Retired	9 (5.8)	1 (11.1)	10 (4.5)	3 (30)
	Others	14 (9.1)	1 (7.1)	12 (5.4)	1 (8.3)
Marital status	Never married	32 (20.8)	0 (0.0)	12 (5.4)	0 (0.0)
	Currently married	122 (79.2)	11 (9.0)	187 (84.2)	2 (6.4)
	Divorced/widowed	0 (0)	0 (0)	23 (10.4)	4 (17.4)
Educational level completed	No school - primary school uncompleted	18 (11.7)	0 (0)	45 (20.3)	1 (2.2)
	Primary school	40 (26.0)	1 (2.5)	69 (31.1)	5 (7.3)
	Lower secondary school	40 (26.0)	0 (0)	58 (26.1)	6 (10.3)
	Higher secondary school - university	56 (36.4)	10 (17.9)	50 (22.5)	4 (8.0)
Monthly household income (USD^b^)	< 71	19 (12.3)	0 (0)	38 (17.1)	4 (10.5)
	71–141	35 (22.7)	3 (8.6)	52 (23.4)	4 (7.7)
	142–283	53 (34.4)	3 (5.7)	70 (31.5)	4 (5.7)
	≥ 284	47 (30.5)	5 (10.6)	62 (27.9)	4 (6.5)
Level of daily physical activity	Heavy	98 (63.6)	3 (3.1)	78 (35.1)	4 (5.1)
	Light	56 (36.4)	8 (14.3)	144 (64.9)	12 (8.3)
Smoking status	Never smoker	31 (20.1)	2 (6.5)	211 (95.1)	16 (7.6)
	Ex-smoker (currently not smoking)	31 (20.1)	3 (9.7)	10 (4.5)	0 (0)
	Current smoker	92 (59.7)	6 (6.5)	1 (0.5)	0 (0)
Alcohol consumption	< 1 day per week - never consumed	83 (53.9)	6 (7.2)	218 (98.2)	16 (7.3)
	1–3 days per week	50 (32.5)	5 (10)	3 (1.4)	0 (0)
	4–7 days per week	21 (13.6)	0 (0)	1 (0.5)	0 (0)
BMI^c^	Low or normal	132 (85.7)	7 (5.3)	180 (81.1)	9 (5.0)
	Overweight	19 (12.3)	3 (15.8)	34 (15.3)	4 (11.8)
	Obesity	3 (2.0)	1 (33.3)	8 (3.6)	3 (37.5)
Hypertension^d^	No	96 (62.3)	3 (3.1)	173 (77.9)	8 (4.6)
	Yes	58 (37.7)	8 (13.8)	(22.1)	8 (16.3)
Hyperlipidemia^e^	No	127 (82.5)	6 (4.7)	190 (85.6)	13 (6.8)
	Yes	27 (17.5)	5 (18.5)	32 (14.4)	3 (9.4)
Metabolic syndrome^f^	No	98 (63.6)	2 (2.0)	196 (88.3)	8 (4.1)
	Yes	56 (36.4)	9 (16.1)	26 (11.7)	8 (30.8)

^a^DM, Diabetes mellitus. Any of the following factors: 1) elevated HbA1c level ≥ 6.5%; 2) elevated fasting plasma glucose (FPG) level ≥ 7.0 mmol/L or random elevated plasma glucose level ≥ 11.1 mmol/L; or 3) history of treatment for DM.

^b^Converted according to the rates in December 2014 (1,000,000 VND = 47.3 USD).

^c^BMI, Body mass index. Overweight (BMI ≥ 25 kg/m^2^ and <30 kg/m^2^), and obese (BMI ≥ 30 kg/m^2^).

^d^Raised blood pressure (BP): systolic BP ≥ 140 mmHg and/or diastolic BP ≥ 90 mmHg.

^e^Any of the following factors: 1)High total cholesterol (TC) level as ≥6.2 mmol/L; 2) low high-density lipoprotein cholesterol (HDL-C) level as <1.0 mmol/L; and 3) high triglyceride level as ≥2.3 mmol/L.

^f^Any three of the following five factors: 1) waist circumference ≥ 90 cm for males or ≥80 cm for females in the Asian population; 2) triglyceride level ≥ 1.7 mmol/L; 3) HDL-C level < 1.0 mmol/L for males and <1.3 mmol/L for females; 4) systolic BP ≥ 130 mmHg and/or diastolic BP ≥ 85 mmHg; and 5) FPG level ≥ 5.6 mmol/L or drug treatment for elevated glucose level.

### Prevalence of DM

Among the 376 participants, 27 were identified as having DM, which accounted for 7.2% (95%CI: 4.6–9.8) of the sample, with 7.1% (3.0–11.3) for males and 7.2% (3.8–10.6) for females. These 27 cases met the criteria for DM, as defined above, as follows: 1) elevated HbA1c level (18/376, 4.8%); 2) elevated FPG level or random elevated plasma glucose level (14/376, 4.0%); and 3) presence of history of treatment for DM (10/376, 2.7%). One case with suspected hemoglobinopathy with extremely high HbA1c level (14.1%) and normal FPG level (4.9 mmol/L) was excluded.

### Risk factors associated with DM and other related conditions

Multivariate analysis identified that those aged 60–70 years (aOR: 8.7; 95%CI: 1.4–56.0) and those being classified as obese according to BMI (aOR: 10.2; 95%CI: 2.2–50.2) were at a higher risk for DM than those aged 30–39 years and those classified as having low or normal BMI, respectively (Table 2).

**Table 2:** Risk factors associated with diabetes mellitus in Khanh Hoa, Vietnam

Factor	Category	Univariate analysis (*n* = 376)	Multivariate analysis (*n* = 302)
		OR (95%CI)^a^	aOR (95%CI)^a^
Sex	Female	ref.	ref.
	Male	1.0 (0.5–2.2)	1.3 (0.3–6.8)
Age (years old)	20–29	-^b^	-^b^
	30–39	ref.	ref.
	40–49	3.5 (0.7–17.1)	2.8 (0.5–15.3)
	50–59	4.6 (0.95–21.7)	3.5 (0.7–18.7)
	60–70	10.9 (2.2–54.4)	8.7 (1.4–56.0)
Residential area	Rural	ref.	ref.
	Urban	3.5 (1.4–9.1)	2.6 (0.8–7.9)
Occupation	Manual worker	ref.	ref.
	Office worker	2.0 (0.5–8.6)	1.3 (0.2–8.8)
	Service worker	2.7 (0.8–8.6)	0.6 (0.1–3.6)
	Housekeeper, student, no job	2.0 (0.6–6.6)	0.6 (0.1–3.7)
	Retired	6.5 (1.7–25.8)	0.5 (0.1–4.5)
	Others	2.0 (0.4–10.7)	1.3 (0.2–10.1)
Level of daily physical activity	Heavy	ref.	ref.
	Light	2.7 (1.1–6.5)	2.1 (0.5–8.8)
Smoking status	Never smoker	ref.	ref.
	Ex-smoker (currently not smoking)	1.0 (0.3–3.5)	0.7 (0.1–4.5)
	Current smoker	0.9 (0.3–2.2)	0.7 (0.1–4.3)
BMI	Low or normal	ref.	ref.
	Overweight	2.8 (1.1–7.2)	1.2 (0.4–3.7)
	Obesity	10.6 (2.8–39.9)	10.2 (2.2–50.2)
Hypertension	No	ref.	ref.
	Yes	4.1 (1.7–10.2)	2.0 (0.8–5.2)
Hyperlipidemia	No	ref.	ref.
	Yes	2.5 (1.02–5.9)	2.8 (0.9–8.7)

*OR* odds ratio, *aOR* adjusted odds ratio, *CI* confidence interval, *ref.* reference, *BMI*, body mass index

^a^Calculated using logistic regression

^b^No cases of DM existed for analysis

### Perception and self-reported history of DM

More than two-thirds (254/376, 67.6%) of the participants either did not understand or had never heard of DM, and less than half of the DM cases (12/27, 44%) had been aware of or reported their history of DM (Table 3).

**Table 3:** Perception and self-reported history of diabetes mellitus in Khanh Hoa, Vietnam

Factor	Category	Total n (%)	DM n (%)
Perception of DM		(*N* = 376)	(*N* = 27)
	NEVER HEARD of it	44 (11.7)	3 (11.1)
	Ever heard of it but NOT UNDERSTAND what it is	210 (55.9)	11 (40.7)
	Ever heard of it and understand what it is	122 (32.5)	13 (48.1)
Current and/or past history of DM		(*N* = 334)	(*N* = 24)
	Yes	36 (10.8)	12 (50.0)
	No	275 (82.3)	12 (50.0)
	Unknown	23 (6.9)	0 (0.0)

*DM* Diabetes mellitus

## Discussion

This study yielded the following three main findings: the prevalence of DM among the adult population in central Vietnam was 7.2% (95% CI: 4.6–9.8), being of older age and classified as obese according to BMI were risk factors associated with DM, and residents in central Vietnam had limited awareness of DM.

The prevalence of DM among the adult population in Khánh Hòa Province was slightly higher than national estimates or findings from other studies in northern and southern areas (3.7–6.0% (95%CI: 2.7–6.7%) [4–9]. The prevalence of overweight/obesity according to BMI was compatible with findings from other studies [5, 10], while those of hypertension for both sexes and metabolic syndrome for males were far higher in this study compared to others [10, 11]. The reasons for these discrepancies remain unknown, but because such potentially crucial health issues could be worrisome, these findings require further investigation.

Being older and classified as obese according to BMI were found to be risk factors for DM, which is consistent with findings from other studies in Vietnam [6–9]. The confidence interval of aOR in multivariate analysis in those aged 60–70 and being obese defined by BMI as associated factors with DM widely ranged. One primary reason for it here would be the smaller sample size, while it could be possibly affected by individual variation in impaired level of glucose tolerance due to resistance or impaired secretion of insulin with age or obesity.

More than two-thirds of the participants either did not understand or had never heard of DM, and less than half had been aware of or reported their history of DM. This finding suggests that advocacy and education regarding preventive measures for DM are required to encourage behavioral change for reducing risk factors and promoting the seeking of medical assistance when necessary, as well as consolidation of the health system to ensure proper medical care for DM and other noncommunicable diseases.

This study did have several limitations. First, the response rate was lower than expected before the survey (78.3% vs. 95%). This lower response rate could have introduced a bias regarding the prevalence of DM, and care should therefore be taken when interpreting the results. Second, under the logistical constraints, HbA1c and FPG were used to diagnose DM instead of the oral glucose tolerance test recommended by the WHO [16]; these tests could be a practical option as a feasible method for detecting DM in seroprevalence surveys in settings with limited resources. Third, hemoglobinopathy, which is a relatively prevalent disorder in Southeast Asia, can compromise the consistency of results for DM diagnosis because it affects HbA1c levels [22, 23]. Therefore, in this study, we excluded one suspected case that demonstrated apparent discordance between HbA1c and FPG levels without laboratory testing for confirmation.

Regardless of these limitations, this population-based study revealed a general epidemiological picture of DM in central Vietnam, and the results are expected to provide insight on affected populations and actions to be prioritized when developing a national program for the prevention of DM. The methods we used in this study also demonstrated good applicability and feasibility of the methods, including PPS sampling or HbA1c for diagnosis of DM, as promising options for future field surveys in resource-limited settings.

## Conclusions

The prevalence of DM among the adult population in central Vietnam was slightly higher than that in other areas of Vietnam. Furthermore, those of an older age and classified as obese according to BMI were found to be at higher risk for DM. Additional studies are needed to further explore perceptions and practices regarding DM in such populations.

## Abbreviations

aOR: Adjusted odds ratio
BMI: Body mass index
BP: Blood pressure
CHCs: Commune health centers
CI: Confidence interval
DM: Diabetes mellitus
EDTA: Ethylenediaminetetraacetic acid
FPG: Fasting plasma glucose
HbA1c: Glycosylated hemoglobin
HDL-C: High-density lipoprotein cholesterol
HPLC: High-performance liquid chromatography
IPN: Pasteur Institute Nha Trang
NCGM: National Center for Global Health and Medicine
OR: Odds ratio
PPS: Pprobability proportional to size
STEPS: STEPwise approach to noncommunicable disease risk factor surveillance
TC: Total cholesterol
WHO: World Health Organization
